# Deep learning system for true- and pseudo-invasion in colorectal polyps

**DOI:** 10.1038/s41598-023-50681-5

**Published:** 2024-01-03

**Authors:** Joe Yang, Lina Chen, Eric Liu, Boyu Wang, David K. Driman, Qi Zhang, Charles Ling

**Affiliations:** 1 Department of Computer Science, Western University, London, N6A 3K7 Canada; 2 Department of Pathology and Laboratory Medicine, Western University, London, N6A 3K7 Canada; 3 Sunnybrook Health Sciences Centre, University of Toronto, Toronto, M4N 3M5 Canada

**Keywords:** Colon cancer, Computer science, Software

## Abstract

Over 15 million colonoscopies were performed yearly in North America, during which biopsies were taken for pathological examination to identify abnormalities. Distinguishing between true- and pseudo-invasion in colon polyps is critical in treatment planning. Surgical resection of the colon is often the treatment option for true invasion, whereas observation is recommended for pseudo-invasion. The task of identifying true- vs pseudo-invasion, however, could be highly challenging. There is no specialized software tool for this task, and no well-annotated dataset is available. In our work, we obtained (only) 150 whole-slide images (WSIs) from the London Health Science Centre. We built three deep neural networks representing different magnifications in WSIs, mimicking the workflow of pathologists. We also built an online tool for pathologists to annotate WSIs to train our deep neural networks. Results showed that our novel system classifies tissue types with 95.3% accuracy and differentiates true- and pseudo-invasions with 83.9% accuracy. The system’s efficiency is comparable to an expert pathologist. Our system can also be easily adjusted to serve as a confirmatory or screening tool. Our system (available at http://ai4path.ca) will lead to better, faster patient care and reduced healthcare costs.

## Introduction

Colon cancer ranks as the third most frequently occurring type of cancer in North America^1^. Up to 50% of adults over 50 years of age harbor at least one colorectal polyp. While most of them are benign, some of those polyps can be cancerous. Colonoscopies are a procedure that is routinely performed on adults to examine the colon to find abnormalities. Over 15 million colonoscopies are performed yearly in North America. During a colonoscopy, abnormal colon polyps are extracted to make into tissue slides for examination by pathologists. Distinguishing between true- and pseudo-invasion in colon polyps is critical in treatment planning^2^. Surgical resection of the colon is often the treatment option for true invasion, whereas long-term monitoring is recommended for pseudo-invasion.

It could be extremely challenging, however, to distinguish true- and pseudo-invasion for the following reasons. First of all, the features used to differentiate true- and pseudo-invasion can be very subtle and complex. Recognition of distorted structures and complex morphological features often requires a panel of pathologists. Secondly, the slides inspected under microscopes are frequently large, and pathologists often need to use varying zoom levels to locate only a few crucial areas.

**Figure 1:**
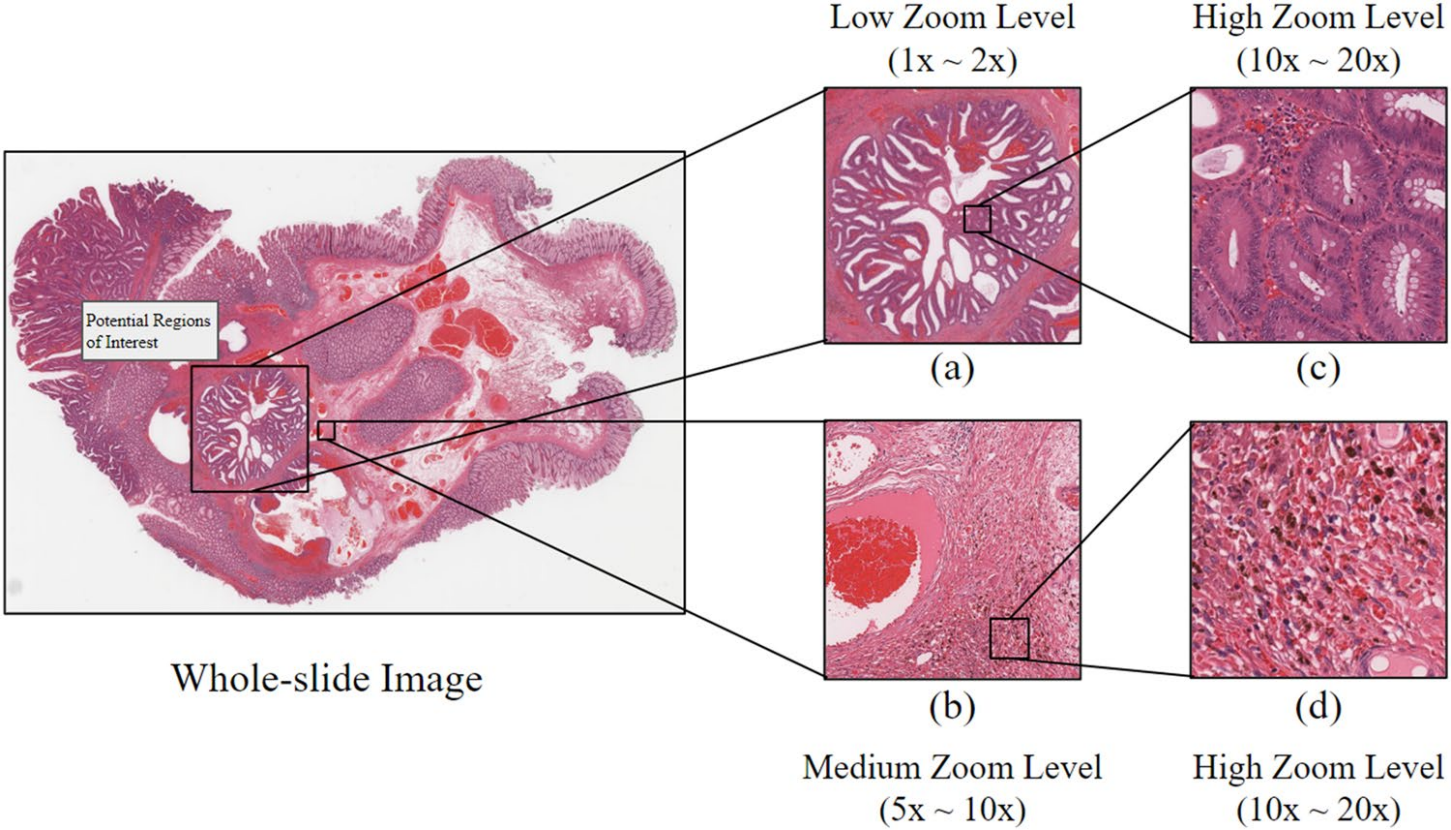
An example of pathologists’ whole-slide image examination workflow under different magnifications (zoom) levels. Pathologists typically commence their examination of a whole-slide image by identifying probable regions of interest at low or moderate magnifications. Subsequently, they zoom into the regions of interest at higher magnifications for further examination. Based on their observations at different zoom levels, they establish whether the whole-slide image portrays true- or pseudo-invasion.

As shown in Fig. 1, typically, pathologists examine tissue slides at low magnification (zoom) level (e.g. $1\times$ to $2\times$) to identify regions of interest first. They then zoom in on each interested area to gather more detailed evidence, which may involve analyzing certain types of cells, tissue structures, stromal features, or interaction of groups of cells. High magnifications (e.g. $10\times$ to $20\times$) are necessary to visualize individual cells. Spatial relations are also important. Challenging cases often need to be reviewed by one or more expert pathologists. The entire process may take days to complete.

With the development of digital pathology, transforming histological slides into highly detailed whole-slide images (WSIs) through scanning has become popular. Furthermore, deep learning, being one of the most powerful techniques of artificial intelligence, also has vast applications in digital pathology. Nevertheless, utilizing deep learning for true- and pseudo-invasion classification on WSIs is still considerably challenging. Firstly, the typical size of one WSI is enormous (averagely around 100,000 by 100,000 pixels), which is equivalent to tens of thousands of ordinary medical images. Additionally, to the best of our knowledge, there is currently no labeled dataset available to train our deep-learning models. Moreover, the process of labeling WSIs is exceedingly labour-intensive. To deal with this difficulty, we developed an innovative system for annotating tissue types across various (e.g. $1\times$ to $40\times$) magnifications. Although it requires some professional knowledge and time for annotating, the demand for labeled images is significantly reduced. To train our models, we acquired 150 cases from London Health Science Center (LHSC). However, only 50 of those cases have been annotated. As a result of the aforementioned difficulties, there currently exists no dedicated software tool for this crucial task. Although some prior endeavors^3–10^ have been undertaken, they can not accomplish the tasks we have. Most of the current works^8–10^ on colon polyp detection and classification focus on colonoscopic images, which are profoundly different from whole-slide histological images in terms of image size, colour, feature and the volume of information they contain. As a result, methods that are effective for colonoscopic colon polyp detections are generally not transferable to our task. On the other hand, most of the existing methods for whole-slide histological image analysis^3–7^ follow a patch-based procedure at a single zoom level. On small patches, the cytological atypia is the most common information generated and used for diagnosis. However, diagnosis of adenocarcinoma requires not only cytological features but also architectural and stromal findings. In fact, the features useful for the differentiation of invasive adenocarcinoma from high-grade dysplasia are not cytological atypia but desmoplastic stroma. These tissues containing architectural and stromal information vary in size and may be too large to fit into the patches or too small to be identified at one fixed zoom level.

In our work, we designed and implemented a novel deep-learning system, consisting of multiple deep neural networks. Our approach was inspired by the multi-zoom-level examination procedure employed by pathologists (as illustrated in Fig. 1), and to mimic pathologists’ workflow. We first partition the WSIs into patches at low, medium, and high magnification (zoom) levels. See Fig. 3 for an overview of our design. Thereafter, for tissue type recognition, three convolutional neural networks (CNNs) were trained to classify the patches at their respective magnifications, solving the unbalanced model performance issue. Besides, we designed an additional CNN to aggregate the tissue type recognition results into a final true-/pseudo-invasion result, as shown in Fig. 5.

**Figure 2:**
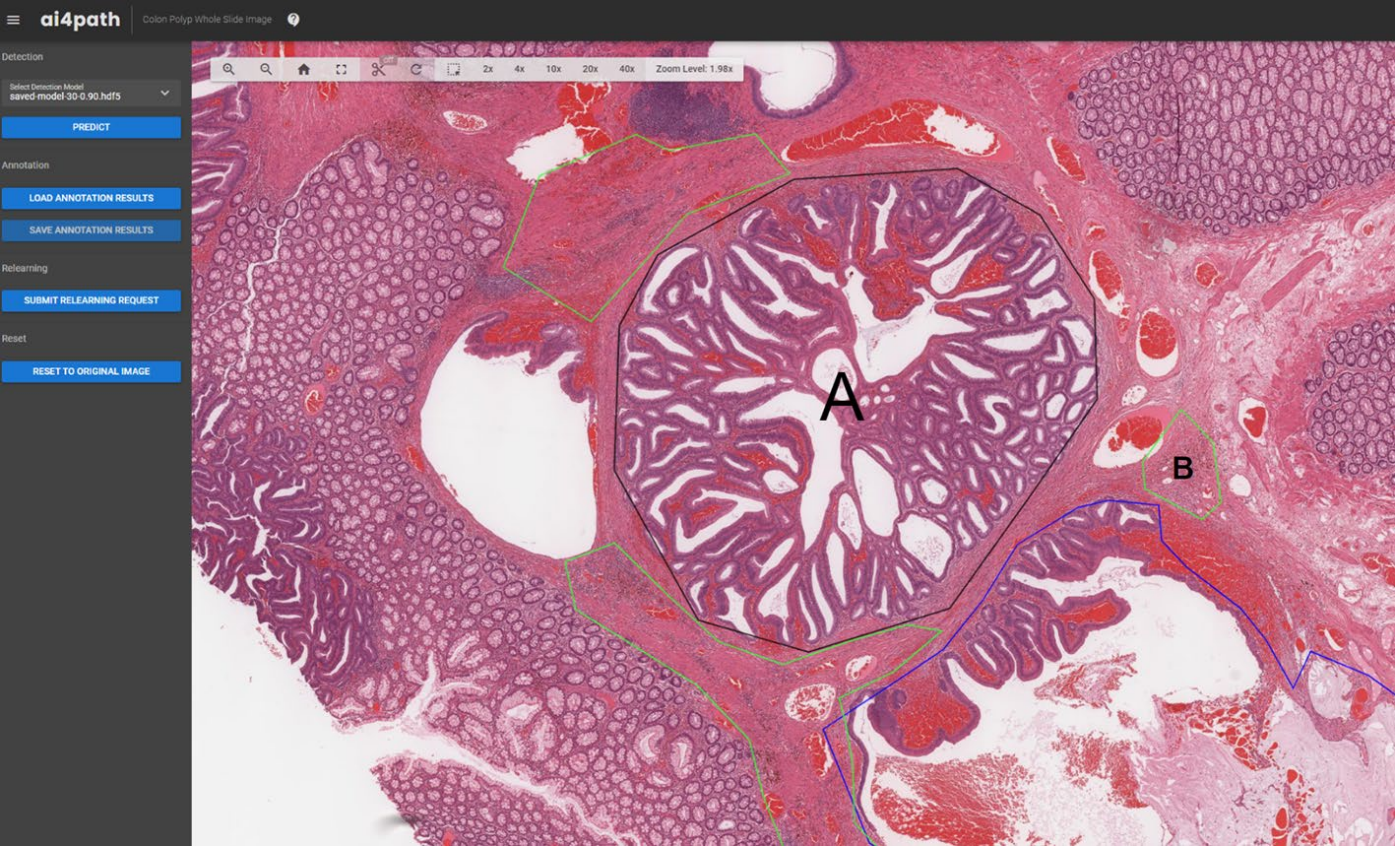
An example of a pathologist annotated WSI using our developed annotation tool. Area A and B correspond to the regions of interest (**a**) and (**b**) shown in Fig. 1. The pathologists annotated the regions of interest by outlining the regions and giving them names.

To facilitate annotation as well as prediction, we have developed a web-based system (AI4Path) specifically for pathologists. See Fig. 2 for the actual annotation. Unlike other digital pathology applications, AI4Path requires no specialized knowledge to operate. It is also easily accessible on any modern computer due to its web browser-based platform. Moreover, AI4Path provides the flexibility of customizable subclass sensitivity, making it easily adjustable for confirmation (as a second opinion) or screening (with minimum false negative errors). For details, see Fig. 7. With the screening function, our system can automatically detect suspicious cases, thus reducing pathologists’ workload. With the confirmatory function, our system can act as a reliable expert pathologist to support primary pathologists in community hospitals. This significantly reduces the turnaround time of pathology reports and minimizes out-of-institution consultations, lowering healthcare costs.

According to the results of our study, our novel system presents a remarkable accuracy rate of 95.3% in classifying tissue types and an impressive accuracy of 83.9% in distinguishing true- and pseudo-invasions. These high accuracy rates and visualization (see Fig. 8) demonstrate the effectiveness of our system in aiding pathologists in their diagnosis and decision-making process.

AI4Path is currently being clinically tested and used in pathology labs at Western University and the University of Toronto. During such utilization, additional WSIs and annotations are collected for the training of the deep neural networks to further improve accuracy. AI4Path has been offering the prospect of a cost-effective and convenient pathology tool. We expect that our innovative and efficient system will help diminish the workload of pathologists and relieve the financial strain on the public health system.

Looking ahead, we are planning to initiate a stage-two study that aims to expand the scope of AI4Path’s applications and test its robustness across a more diverse set of samples. This upcoming phase will involve a wider range of pathologist participation in terms of data collection and real-life user feedback. This will enable us to harness more holistic feedback and insights. By doing so, we aspire to achieve higher precision in our tool and to ensure that it’s well-suited to cater to the evolving needs of the medical community. Through this stage-two study, we also aim to solidify AI4Path’s position as a game-changer in modern pathology practice.

It is also noteworthy to mention that this paper extends the work presented in an abstract^11^, which was published during the 112th Annual Meeting of the United States and Canadian Academy of Pathology (USCAP) in March 2023.

## Methods and materials

### Research ethics

This study was approved by the Western University Research Ethics Board (HSREB 116725). The requirement to obtain informed consent was waived by the Western University Research Ethics Board for the following reasons: the information used in this study is non-identifiable and not obtaining consent is unlikely to adversely affect the welfare of individuals to whom the information is related. All methods in this study were conducted in accordance with relevant guidelines and regulations.

### Study cohort and digital pathology image acquisition

A retrospective case search was performed at the London Health Sciences Centre (LHSC). A total of 150 cases of colorectal polyps with true invasion and pseudo-invasion were selected. All cases were diagnosed with consensus by at least three of the seven GI experts on our pathology review panel. The hematoxylin & eosin (H &E) stained slides were scanned by an Aperio CS scanner for high-resolution WSIs. The diagnosis of pseudo-invasions was based on the presence, location and shape of specific types of tissue on the WSIs, which were annotated for AI algorithm development. Further details on tissue types and annotations are provided in the subsequent “Datasets” section.

### Datasets

In our study, we employed two datasets to train and validate our models. The first dataset consists of 150 LHSC cases. Among these, 67 WSIs were labelled as true invasions, while the remaining 83 WSIs were labelled as pseudo-invasions. Two expert pathologists were involved in the WSI annotation process as WSI annotation requires expert knowledge. Pathologists randomly selected and annotated only 25 true invasion slides and 25 pseudo-invasion slides because the annotation process was extremely labour-intensive. All annotations were done using our developed annotation tool. Figure 2 presents an example of a WSI annotated by pathologists using our developed annotation tool. These annotations included nine categories^12,13^ that pathologists believe are features differentiating true versus pseudo-invasions, namely acellular mucin, angulated gland, desmoplastic stroma, hemorrhage, lamina propia type stroma, luminal necrosis, mucus lake with peripheral dysplastic glands, rounded lobular group, and stroma with hemosiderin . Each annotation was represented by a tissue name associated with an ordered list of polygon vertex coordinates. However, the annotated tissue categories were not balanced, as some tissues naturally appear more frequently than others in number and size. We converted the annotated areas into collections of $224 \times 224$ image patches at different zoom levels, and discarded any image patch that contains less than 60% of annotated areas.

The second dataset we used was the NCT-CRC-HE-100K^14^ dataset, which is a public dataset collected by the National Centre of Tumor Disease at Heidelberg. We used this dataset merely as a pre-training dataset for self-supervised learning to improve the performance of our models. This dataset is widely used for deep learning applications on colon cancers and tissue type classifications. It includes 100,000 non-overlapping image patches manually taken from 86 H &E stained WSIs at 20x zoom level (0.5 microns per pixel). All images in the dataset were RGB images with dimensions of $224 \times 224$, and they were normalized using Macenko’s image color normalization method^15^. The NCT-CRC-HE-100K dataset includes nine different tissue categories related to colon cancers. However, our pre-training process did not use the category labels from this dataset, as the self-supervised learning method we used did not require image labels as inputs for learning representations.

### System architecture

**Figure 3:**
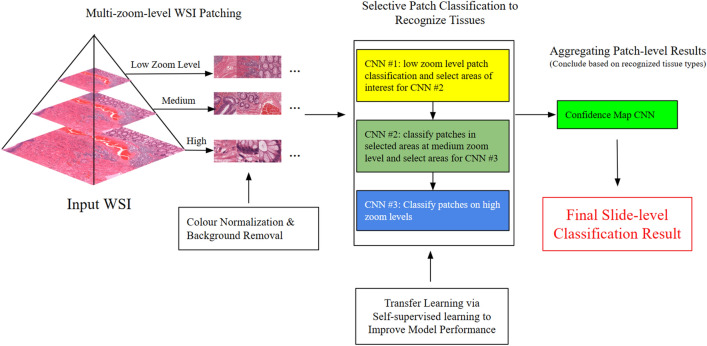
An overview of our novel true- and pseudo-invasion differentiation system, which consists of three main stages: 1. multi-zoom-level patching and patch preprocessing; 2. tissue recognition through selective patch classification; 3. concluding based recognized tissue types by aggregating patch-level results.

Our system is an end-to-end system that comprises three main components, as depicted in Fig. 3. We first patched the input WSI at three different zoom levels and apply color normalization and background removal. Then, we employed three CNNs to recognize tissue types by selectively classifying the image patches into different tissue categories. We improved the performance of these CNNs by transfer learning via self-supervised learning. Third, we designed a shallow CNN that aggregates the patch-level predictions into a slide-level result, which determines whether a WSI contains true invasion or pseudo-invasion based on tissue type recognition.

#### Multi-zoom-level patching

The first part is multi-zoom-level patching, where an input WSI is partitioned into a collection of image patches of size $224\times 224$ pixels at three different zoom levels: $1.25\times$, $10\times$, and $20\times$. To prepare the image patches for further processing, we normalize the RGB colors to a mean of [0.485, 0.456, 0.406] and a variance of [0.229, 0.224, 0.225]. Additionally, we perform simple background removal on each patch by examining its colors and discarding any patch with an average of RGB values close to 1 (e.g. white patches).

#### Selective patch classification

**Figure 4:**
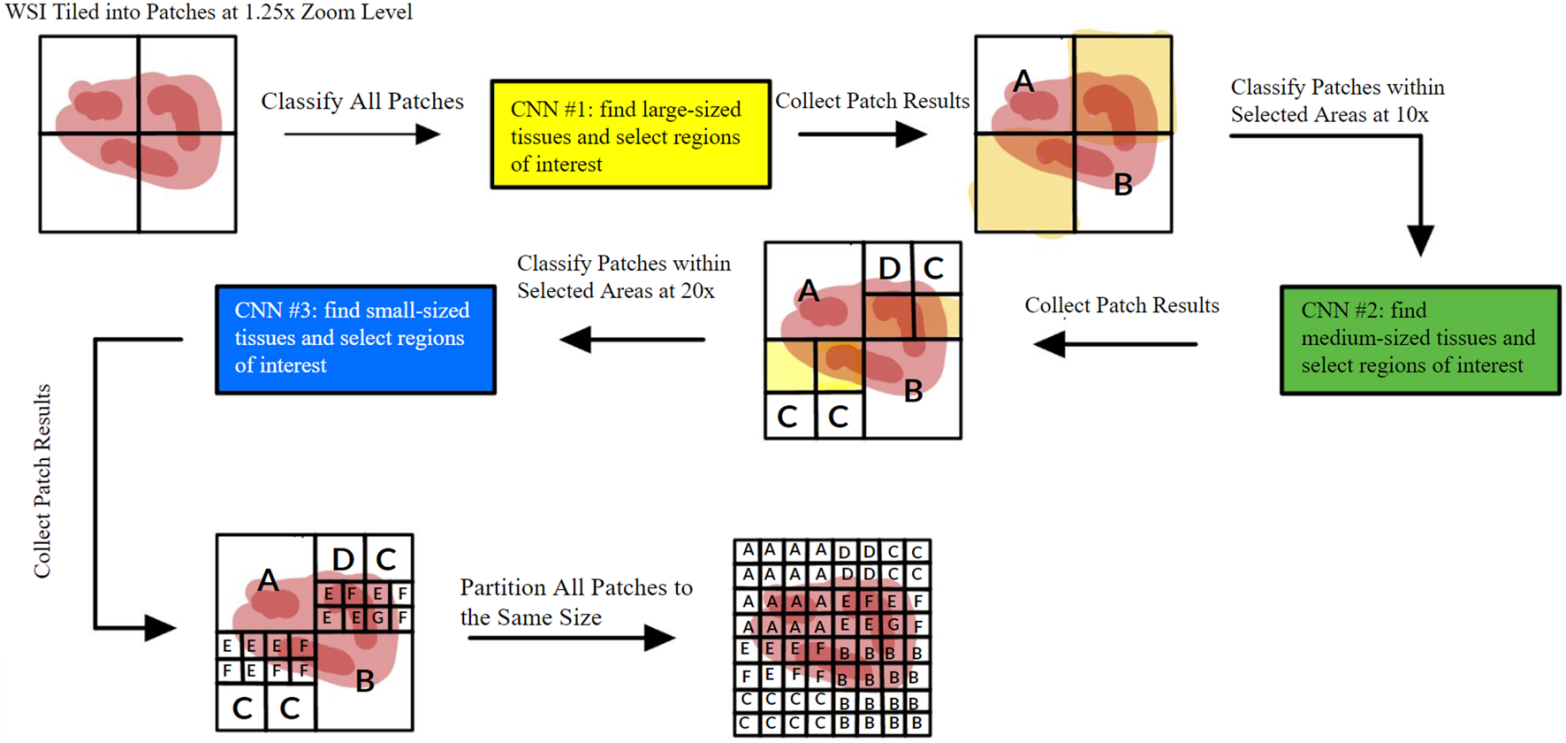
A detailed illustration of our novel selective multi-zoom-level patch classification method for tissue type recognition. Areas of interest are represented in yellow colour. Our method views a WSI by patches. At each zoom level, a CNN recognizes certain tissue types on each patch and selects patches of interest for examination at higher zoom levels.

In the second part of our system, we developed a novel selective multi-zoom-level patch classification method, as shown in Fig. 4, which was inspired by the WSI inspection workflow of pathologists. Pathologists usually start with an overview of the WSI at a very low magnification to identify regions of the lesion, then zoom in on regions of interest to find more evidence. Finally, they conclude based on all the evidence and important features. To mimic this procedure, we trained three CNN using the ResNet-18^16^ architecture to represent each zoom level. The procedure starts by classifying the WSI patches using the first CNN model at $1.25\times$ to find large-sized tissues, such as rounded lobular group and mucus lake, and to select patches of interest for examination at a medium zoom level. Then, the second CNN model was used on the selected patches of interest at a $10\times$ zoom level to find medium-sized tissues and select patches of interest for examination at the highest zoom level. Finally, we used the third CNN model on the selected patches to find small-sized tissues, such as LP stroma and luminal necrosis, at a $20\times$ zoom level. After we obtained all the patch-level results from each level, we partitioned all the patches to the same size and created a 2D map that can be used for future aggregation. This multi-zoom-level design also solved the issue of improper zoom level. The issue usually occurs on single-zoom-level patch-based recognition methods, when the model tries to recognize tissues too large to fit into the current image patch or too small to be identified. This issue will lead to an unbalanced per-class recognition accuracy. Combined with other factors such as data imbalance and per-class learning difficulty, the final model performance might be severely impacted.

One might question how our models select patches of interest for later models. The selection was achieved by training the models to classify all nine tissue categories initially but only keep the predictions of the desired classes, leaving all other predicted patches as patches of interest for later models. However, as the latter models in this workflow only classify regions of interest selected by the previous models, any errors made by previous models have the potential to accumulate and negatively impact the final output accuracy. To address this issue, we established a threshold to accept classification results with only high confidence. Results with low predicted confidence have a high probability of being incorrect, and we excluded them to reduce the risk of error propagation. Subsequently, the later models in the pipeline will re-examine regions with low classification confidences at other zoom levels, increasing the likelihood of achieving accurate results.

#### Aggregating patch-level results

**Figure 5:**
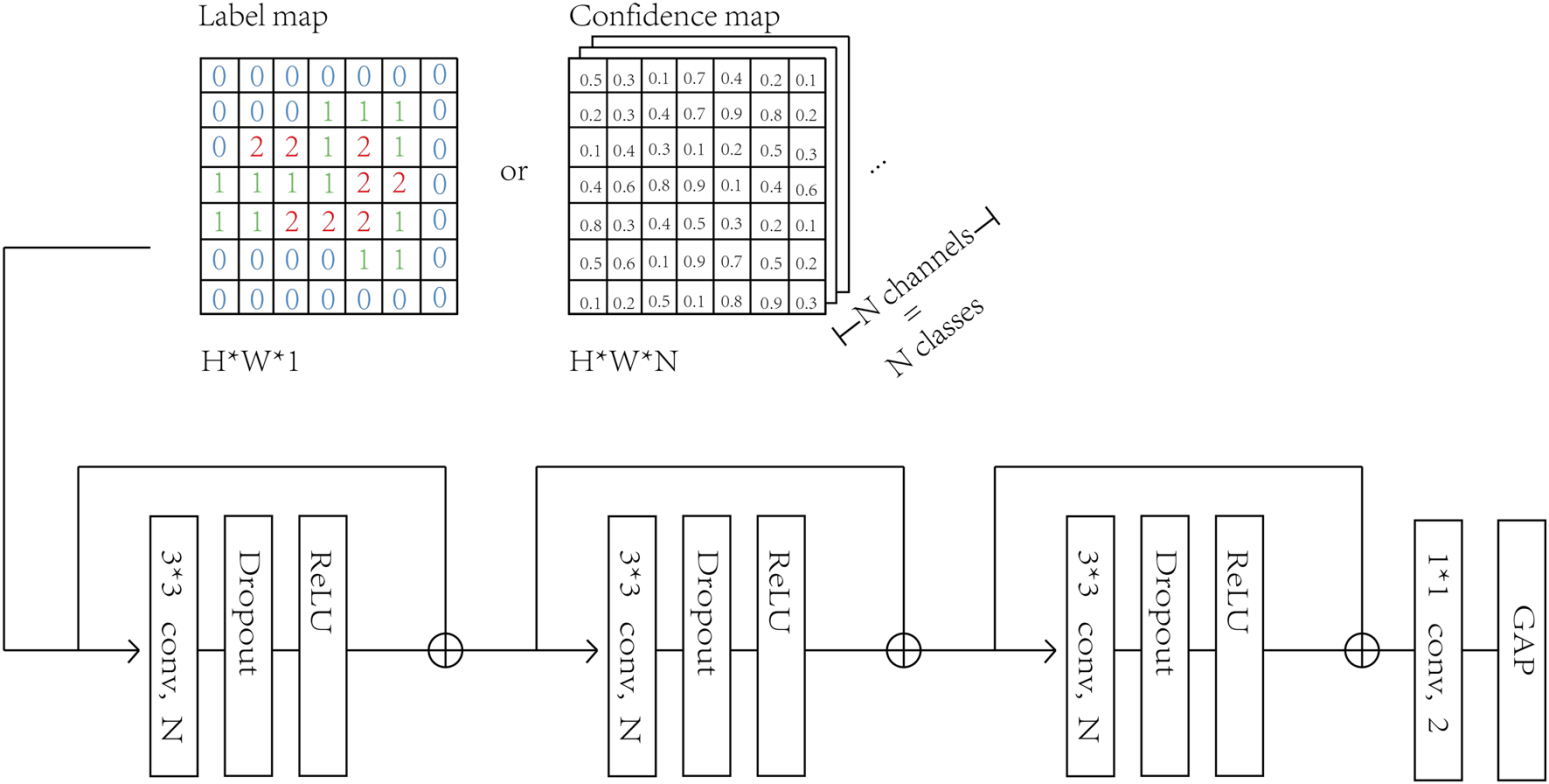
Architecture of the designed CNN for aggregating patch-level results into a slide-level result. The CNN consisted of three main convolutional blocks and an output block. The CNN was designed to be shallow so that the model complexity does not overwhelm the small size of our dataset. With more training samples, we can increase the model complexity by adding more layers and using more complicated designs.

In previous studies, linear classifiers were often applied to classify hand-crafted features, such as class percentages, to aggregate patch-level results into slide-level results. However, such linear classifiers ignore the 2D positional relationship between patch results, leading to suboptimal performance. In contrast, we designed a shallow CNN to aggregate the patch-level results by classifying a label map or a confidence map, as depicted in Fig. 5. A label map is a 2-dimensional tensor of size $H \times W$, in which each cell in the matrix stores the label index of the patch in the corresponding location in the WSI. A confidence map stores a list of *N* class confidence values in each cell, creating a 3-dimensional tensor of size $H \times W \times N$. In our experiments, we found that classifying confidence maps always leads to better performance than using label maps. Due to the limited number of WSIs in our dataset, we could not use deep CNN architectures, since the model complexity would be too high compared to the number of training samples and result in poor performance. Thus, we designed a shallow CNN with an appropriate complexity for our dataset, which consists of four convolutional blocks.

Each of the first three blocks contains a $3\times 3$ convolution layer with the same number of output channels as input channels, a dropout layer^17^, and a ReLU activation layer^18^. We introduced skip connections before the input layer and after the output layer of the first three convolution blocks to allow a stronger gradient flow for faster training, similar to the ResNet architecture.

Our network differs from conventional deep CNNs, as we feed the output of the last convolution layer into a special convolution block instead of fully-connected linear layers. This last convolution block consists of a $1\times 1$ convolution layer with two output channels, a global average pooling (GAP) layer^19^, and a softmax activation layer as the final output layer of the entire network. The GAP layer^19^, introduced by Lin et al., was used to reduce model complexity. The GAP layer aggregates the spatial information by averaging the values of pixels in each input channel, which is better suited for 2D inputs than forcing them to become 1D vectors to fit into fully-connected layers. Unlike fully-connected layers, the GAP layer has no trainable parameter, reducing the complexity of the model and preventing overfitting. Moreover, the GAP layer is less sensitive to image translation and rotation than fully-connected layers, as the average value of pixels on a rotated or translated image is always very close to that of the original image. Using a GAP layer allows input confidence maps to preserve their shapes, preventing the risk of changing their labels due to resizing.

Since we used the GAP layer as the final output layer for the binary classification of true invasion and pseudo-invasion, the input to the last layer must have only two channels, each containing information related to one type of invasion. To achieve this, we used a $1\times 1$ convolution layer. Unlike conventional $3\times 3$ or $5\times 5$ convolution kernels, which learn to capture local structural features by considering neighbouring pixels, a $1\times 1$ kernel applies only linear weighting along the channels, reducing the number of parameters and time spent on the convolution operation.

### Transfer learning via self-supervised learning

Previous research^20^ has demonstrated that transfer learning can be a valuable tool for accelerating the training process and improving accuracy, particularly when the available data is limited. However, when we attempted to apply transfer learning using the ResNet model pre-trained on the ImageNet dataset^21^, we did not observe a significant improvement in our training process. This is because the ImageNet dataset consists solely of natural object images markedly different from medical images. As a result, we opted to pre-train our models for tissue type recognition using datasets that closely align with our task, so that transfer learning would yield more effective results.

Although pre-training on a similar source dataset and then fine-tuning the model on the target dataset is the most common transfer learning method, recent works^22–24^ suggest that self-supervised learning (SSL)^25–29^ is more effective, especially when the target dataset contains noise. Our dataset contains noise due to imprecise annotation, as it is difficult and time-consuming to outline the tissue areas accurately. However, given the small size of our own dataset, we cannot generate a sufficiently large collection of image patches for SSL to learn discriminative feature representations. Therefore, we chose to apply self-supervised learning as a pre-training method on the NCT-CRC-HE-100K dataset and then transfer the knowledge by fine-tuning the models on our dataset. During pre-training, only the convolutional layers of our models were trained. Subsequently, we fine-tuned the models on our dataset via supervised training, with all convolutional layers frozen and fully-connected layers re-initialized. Additionally, the output layer was substituted to match the dimension of the number of output classes.

We used Bootstrap-Your-Own-Latent (BYOL)^29^ as the SSL method. This method learns robust representations by contrasting the model with a slightly different version of itself using different views of the same input. Given an input image *x*, and two different augmentations *t* and $t'$, at each iteration, the augmented images *t*(*x*) and $t'(x)$ are passed through two models with parameters $\theta$ and $\xi$, respectively, where $\theta$ is the desired set of parameters, and $\xi$ is the exponential moving average of $\theta$. The exponential moving average of $\theta$ is computed using the following equation:

$$\begin{aligned} \xi \leftarrow \tau \xi + (1 - \tau )\theta \end{aligned}$$

where $\tau \in [0, 1]$ is the decay factor for the exponential moving average. Then, BYOL computes the mean-squared error between the predictions made by the two models, as shown in the formula below:

$$\begin{aligned} L_{\theta , \xi } = || h(\theta , t(x)) - h(\xi , t'(x)) ||^2_2 \end{aligned}$$

where $h(\theta , t(x))$ is the prediction from the model with desired parameters $\theta$ and input augmented by *t*, and $h(\xi , t'(x))$ is the prediction from the model with parameter $\xi$ and input augmented by $t'$. Finally, BYOL updates the parameters $\theta$ by backpropagating the computed loss and starts the next iteration of the same procedure. By augmenting the input differently, the model learns to capture the semantic information of the input image, thus learning representations robust to the views of the same input.

## Results

### Patch-level tissue recognition accuracy

We converted the annotated regions into collections of image patches at the desired zoom levels, which we utilized for training and evaluating our models. To evaluate the models’ performance, we randomly split the collection of image patches, utilizing 80% for training and the remaining 20% for testing. We employed classification accuracy as our evaluation metric, which is defined as the number of correctly classified images divided by the total number of images. The average accuracy of our models was 95.3%.

**Figure 6:**
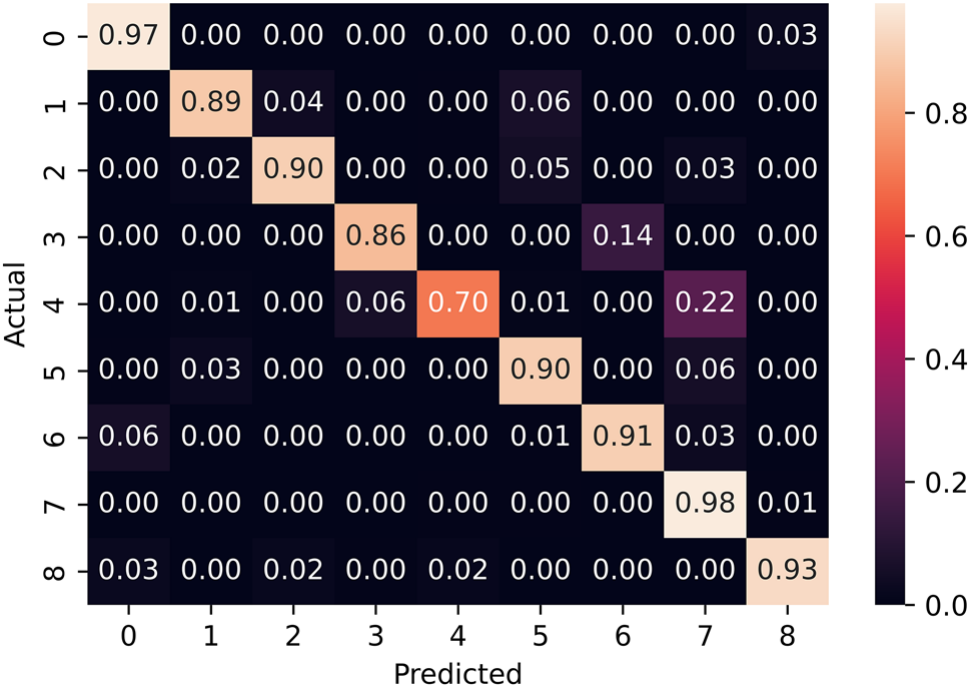
Normalized confusion matrix. The categories are acellular mucin, angulated gland, desmoplastic stroma, hemorrhage, lp stroma, luminal necrosis, mucus lake with peripheral dysplastic glands, rounded lobular group, and stroma with hemosiderin, as ordered on the figure axis. Our models achieved balanced accuracy in every class except for the fifth class (class number four on the figure axis), where we lacked training samples in our dataset.

However, the categories in our dataset were unbalanced. Therefore, the accuracy might not provide enough information. Hence, we analyzed the per-class classification accuracy using a confusion matrix. For a more intuitive interpretation, we normalized the confusion matrix to show percentages of predicted samples instead of exact numbers, as depicted in Fig. 6. The confusion matrix shows that per-class accuracy was mostly balanced, as the models were able to predict most classes with accuracy above 90%. However, we observed that our models did not perform well on the fifth class (class number four on the figure axis), lp stroma. This class lacked annotations in our dataset to generate training samples, resulting in suboptimal accuracy.

### Slide-level classification accuracy

As mentioned in earlier sections, our system obtains slide-level classification results for true- and pseudo-invasion by utilizing a shallow CNN to classify the confidence maps for each WSI. To generate the confidence maps, we utilized our system to produce one confidence map for each WSI in our dataset. We evaluated the performance of our shallow CNN using K-fold cross-validation on these 150 confidence maps. K-fold cross-validation is a widely used method for assessing the performance of a machine-learning model on an independent dataset. The process involves dividing the dataset into K subsets of equal size. The machine learning model is trained K times, with each of the K subsets used exactly once as validation data, while the remaining K-1 subsets are used as training data. The K results are then averaged to produce a single estimation of the model’s performance. For our cross-validation process, we set K = 10, and our shallow CNN achieved an average accuracy of 83.9%.

### Impact of patch prediction confidence threshold

In the second part of our system, we employed a threshold to remove patch predictions with low confidence. This approach is advantageous since low-confidence predictions are more likely to be incorrect. However, setting the correct threshold is critical because a high threshold can remove correct predictions, whereas a low threshold may allow incorrect predictions to be accepted. Thus, we analyzed the impact of the patch prediction threshold on the final true- and pseudo-invasion classification performance. We examined our system’s false-positive (FP) rate and false-negative (FN) rate at each threshold. The FP rate and FN rate are defined as follows::

$$\begin{aligned} & {\text{FP}}\;{\text{rate}} = \frac{{\# \;{\text{of}}\;{\text{False}}\;{\text{Positives}}}}{{\# \;{\text{of}}\;{\text{False}}\;{\text{Positives}} + \# \;{\text{of}}\;{\text{True}}\;{\text{Negatives}}}} = \frac{{\# \;{\text{of}}\;{\text{Incorrect}}\;{\text{Pseudoinvasions}}}}{{\# \;{\text{of}}\;{\text{Incorrect}}\;{\text{Pseudoinvasions}} + \# \;{\text{of}}\;{\text{Correct}}\;{\text{True}}\;{\text{invasions}}}} \\ & {\text{FN}}\;{\text{rate}} = \frac{{\# \;{\text{of}}\;{\text{False}}\;{\text{Negatives}}}}{{\# \;{\text{of}}\;{\text{False}}\;{\text{Negatives}} + \# \;{\text{of}}\;{\text{True}}\;{\text{Positives}}}} = \frac{{\# \;{\text{of}}\;{\text{Incorrect}}\;{\text{True}}\;{\text{Invasions}}}}{{\# \;{\text{of}}\;{\text{Incorrect}}\;{\text{True}}\;{\text{Invasions}} + \# \;{\text{of}}\;{\text{Correct}}\;{\text{Pseudoinvasions}}}} \\ \end{aligned}$$

**Figure 7:**
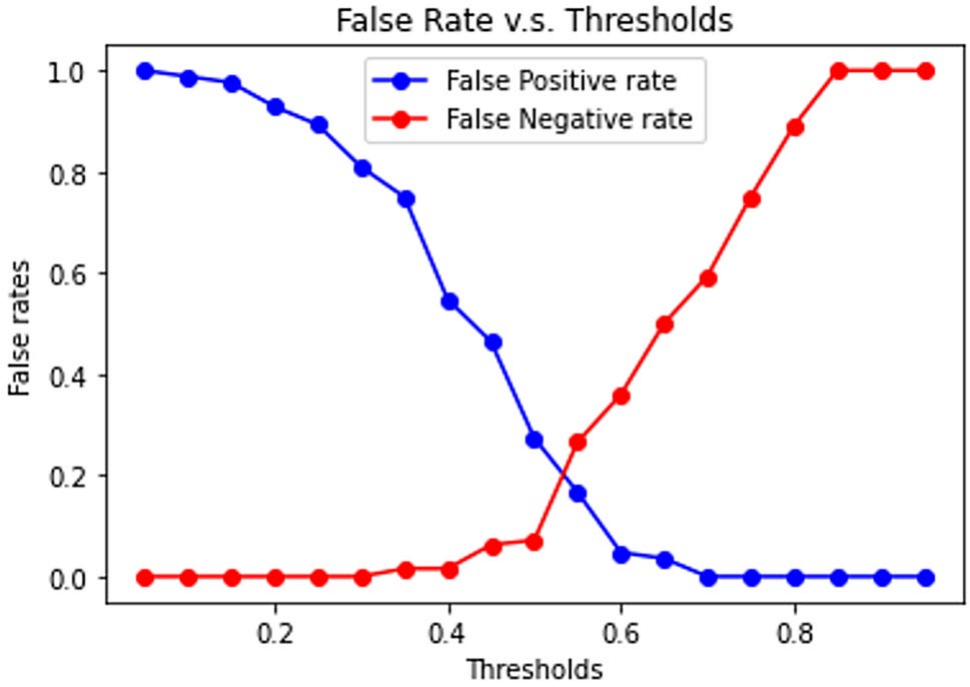
Threshold v.s. False rates. Higher thresholds lead to higher FN rates and lower FP rates, while lower thresholds lead to higher FP rates and lower FN rates.

The FP and FN rates corresponding to each threshold value are illustrated in Fig. 7. We observed that both lines intersect when the threshold equals 0.55, where the system had the most balanced FP and FN rates. Nevertheless, the threshold can be adjusted depending on the situation and requirements. For confirmatory purposes, a high threshold can be set to have a low FP rate, providing a second opinion to the pathologists. On the other hand, for screening purposes, a low threshold can be applied to have a low FN rate, making the system behave like a “stringent” pathologist that assures true invasions are not missed.

### System efficiency

Efficiency is an essential criterion in evaluating the practicality of a method in real-life applications. To assess the efficiency of our system, we randomly sampled ten WSIs from our dataset and calculated the average time taken for our system to recognize tissues and provide slide-level results. Our analysis showed that the system spends an average of 771.5 s (12.8 min) analyzing a WSI, which is comparable to the average time^30^ an expert pathologist would spend examining an image. However, the time a pathologist takes to examine WSIs can vary significantly depending on various factors such as slide complexity, the pathologist’s experience, and the resources available. Pathologists can sometimes spend hours examining ambiguous cases and require cross-checking from colleagues to arrive at a consensus.

It is worth noting that the time taken by our system only depends on the size of tissue areas in a WSI and is insensitive to other factors. Furthermore, our system is currently implemented without utilizing any parallel programming techniques and only runs on a single GPU. We are continuously working on improving the system, and expect to increase the efficiency multiple times if we incorporate such improvements.

### Visualization

**Figure 8:**
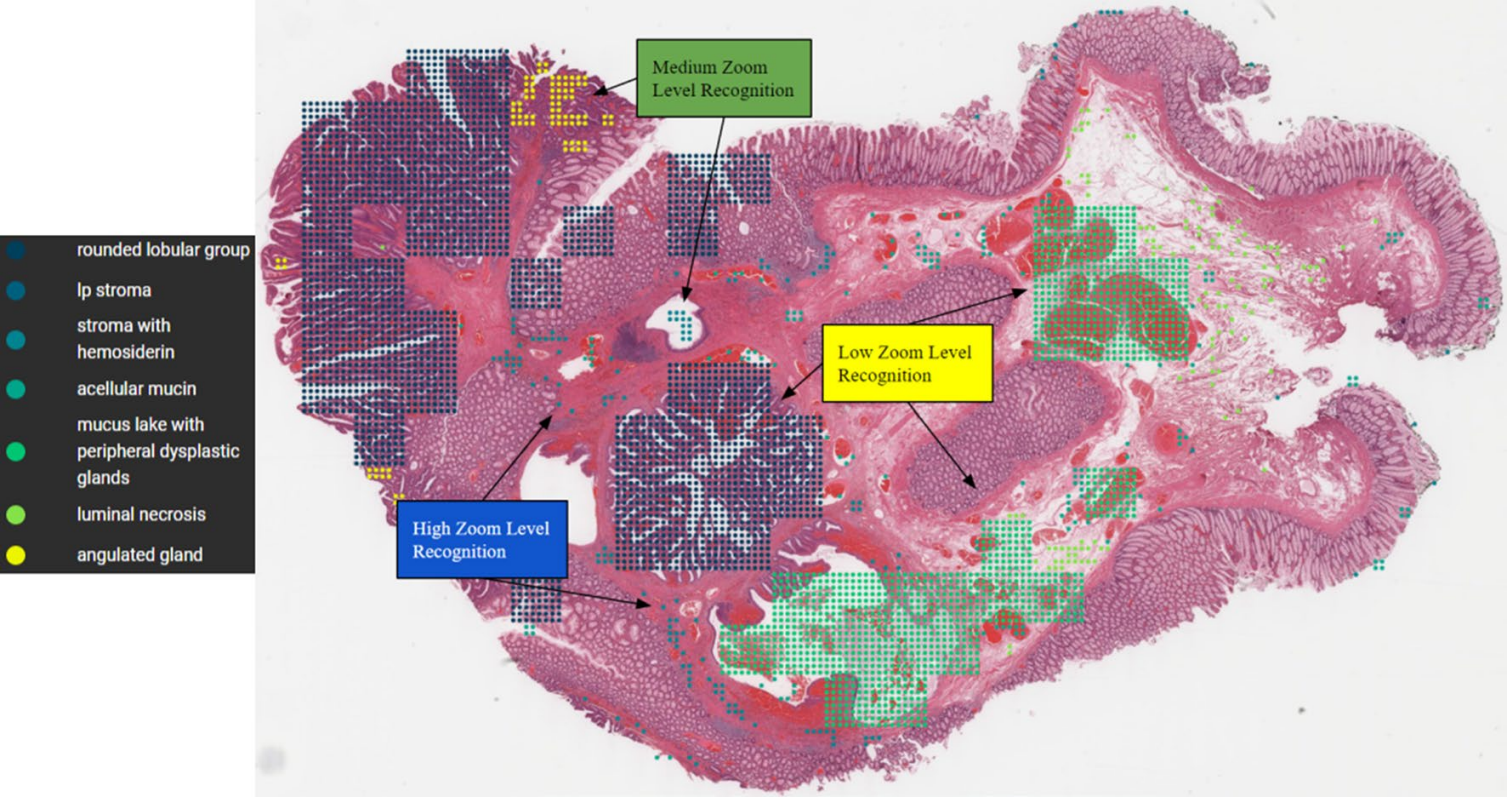
A visualization of the tissue recognition results for a WSI from our system, which can provide visual evidence for pathologists to understand the final slide-level result from our model. Different groups of coloured dots represent different types of tissue areas that are used as evidence to make the final prediction.

Visualizing per-patch results is crucial for human interpretation since the final predictions rely on the tissue type recognition results. In Fig. 8, we present an example of tissue type recognition results visualized on a WSI. Each group of dots corresponds to an area of interest, and each color represents a different tissue type. These per-patch results not only serve as evidence to help pathologists understand the final prediction results but also assist them in identifying areas of interest more efficiently. The visualization also illustrates our multi-zoom-level tissue type recognition procedure, as one can see that the dots are grouped in squares of different sizes.

### Ablation study

One may wonder why we did not opt for direct classification of downsized WSIs or exhaustive recognition of all patches at every zoom level. Both approaches have their respective strengths. The direct classification of downsized WSIs is significantly faster than the classification of tens of thousands of image patches. Exhaustive recognition of all patches at every zoom level and combining the patch-level results at each area ensures a more robust recognition performance. To address these concerns, we compared our method against these approaches, as demonstrated in Table 1. The results reveal that directly classifying downsized WSIs into true and pseudo-invasion results in notably lower accuracy, which is almost the same as taking random guesses for binary classification problems. Exhaustively classifying all image patches at every zoom level and aggregating the information doubles the time required while providing only a minimal increase in accuracy. On the other hand, our approach offers both efficiency and high accuracy. As mentioned earlier, the efficiency of our system can be further improved using many methods.

**Table 1:** A comparison between different methods that one might be interested in regarding accuracy and efficiency. Directly classifying downsized WSIs is very fast, but the accuracy is unacceptable. Exhaustively predicting every patch at every zoom level brings a minimal increase in accuracy but doubles the average time. Our selective multi-zoom-level patch prediction method ensures efficiency without compromising accuracy.

Method	Accuracy (%)	Average time (s)
Directly classifying downsized WSIs	59.7	0.3
Exhaustive multi-zoom-level prediction	84.2	1487.1
Selective multi-zoom-level prediction (ours)	83.9	771.5

**Figure 9:**
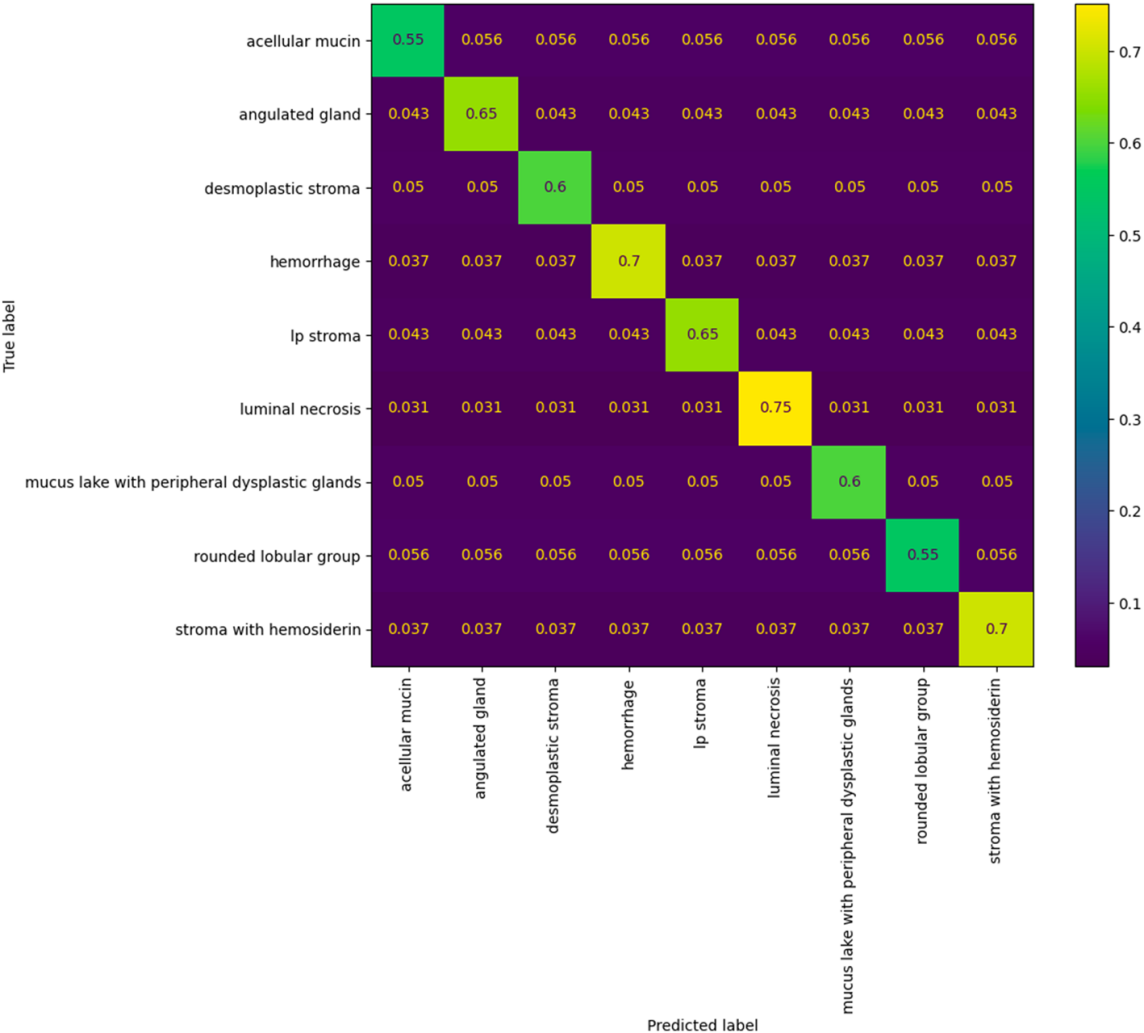
Normalized confusion matrix for U-Net based model experiment.

We also conducted an analysis employing a 224 by 224 sliding window approach on the WSIs to extract training images in the form of patches. Subsequently, we conducted training using a multi-head segmentation model based on the U-Net architecture, which was commonly used in previous works^8–10^. The model produces two primary outputs: a classification type prediction and a 224 by 224 segmentation mask delineating the contour of the tissue of interest. Primarily, the U-Net model, designed for segmentation tasks, posed significant training challenges. This was exacerbated by the limited dataset at our disposal, comprising a mere 50 fully annotated WSIs, resulting in outcomes that fell short of our anticipated standards. Additionally, our proposed method relied on a patch-based strategy, necessitating the application of a 224 by 224 sliding window to traverse each entire whole-slide image. To exemplify this, we conducted a test utilizing a trained U-Net model on one of our vast whole-slide images, measuring 83663 by 76206 pixels. This process generated a total of 126,829 segmented patches, and the inference phase consumed more than 25 min. Upon broader evaluation, the validation accuracy exhibited substantial variance, ranging from 55% to approximately 70%, as depicted in the confusion matrix in Fig. 9. Furthermore, despite the incorporation of an upgraded Nvidia RTX 4090 graphics card, the average inference time remained at approximately 17 min. In direct comparison to our own method, which boasts an average duration of 12.8 min, it is evident that the U-Net segmentation approach not only failed to yield efficiency improvements but also yielded inferior performance results.

## Discussion

Every year, over 15 million colonoscopies are conducted to identify colon cancers, during which the differentiation of true and pseudo-invasion brings the most challenge to pathologists. In our work, we created a novel deep learning system to tackle the arduous task of differentiating true and pseudo-invasion on colon polyp WSIs. Our innovative system possesses several unique advantages that set it apart from previous research. Firstly, our system employs a selective multi-zoom-level per-patch prediction procedure inspired by pathologists, which is both efficient and accurate in tissue type recognition. Secondly, we aggregate the tissue type recognition results while incorporating the spatial relationship between patches, resulting in more dependable slide-level results. Thirdly, our system visualizes the tissue type recognition results through our innovative web-based system (AI4Path), as depicted in Fig. 8.

Moreover, our system offers an adjustable tissue recognition threshold to suit diverse needs. By analyzing the impact of patch confidence thresholds on our final output’s false positive and false negative rates, we have determined the optimal thresholds for different scenarios, such as screening or confirmatory functions. The screening function can alleviate pathologists’ heavy workload. Given that digital pathology has been implemented in numerous pathology centers and is expected to be adopted in all pathology departments in the near future, integrating our system into digital pathology platforms can automatically detect suspicious cases for pathologists to review. The confirmatory function can prove very beneficial for pathologists in community hospitals, particularly in sole practice. When the primary pathologist seeks consensus, and no expert pathologist is available, our system can act as a reliable expert pathologist to aid the primary pathologist. This can significantly reduce the turnaround time of pathology reports and provide patients with prompt reports for next-step management guidance. Moreover, it can reduce healthcare costs by minimizing the number of out-of-institution consultations needed.

Our study demonstrates exceptional accuracy in classifying tissue types and differentiating true- and pseudo-invasions, achieving rates of 95.3% and 83.9%, respectively. Additionally, we have assessed the practicality of our novel system by measuring the time required to analyze a WSI, which indicates an average time of 771.5 s. This is comparable to the speed of an expert pathologist in normal cases and significantly faster than an expert pathologist in challenging cases.

While we are content with the insights gained and the robust solution presented in this study, the limited availability of data still constrains the performance of our system. Merely 150 labeled WSIs are available, among which only 50 were annotated by expert pathologists. Even with such limited resources, our system still achieved satisfactory results. We aim to provide our system as a resource for pathologists to use in their daily practice to obtain more data and enhance the performance of the system over time. Furthermore, the system incorporates a built-in annotation tool that allows pathologists to annotate and modify their areas of interest.

As we interpret these outcomes, it’s imperative to mention our upcoming stage-two study. This next phase is conceived to further the depth of AI4Path’s applications, testing its resilience and adaptability across an even broader array of samples. By incorporating a more extensive cohort of pathologists, we aim to acquire diverse feedback, honing the system’s capabilities. The insights from this subsequent study will be instrumental in shaping AI4Path, ensuring it remains at the forefront of technological advancements in pathology.

In conclusion, we present the potential for an affordable and easily accessible assisting tool for pathologists. We anticipate that our pioneering system will help reduce the workload of pathologists and alleviate the financial burden on the public health system.

## Data Availability

Data from the NCT-CRC-HE-100K are available at https://doi.org/10.5281/zenodo.1214456^14^; data from the London Health Science Centre are available from the corresponding author on reasonable requests, and not publicly available due to patient confidentiality.
